# Salt‐in‐Salt Reinforced Carbonate Electrolyte for Li Metal Batteries

**DOI:** 10.1002/anie.202210522

**Published:** 2022-09-21

**Authors:** Sufu Liu, Jiale Xia, Weiran Zhang, Hongli Wan, Jiaxun Zhang, Jijian Xu, Jiancun Rao, Tao Deng, Singyuk Hou, Bo Nan, Chunsheng Wang

**Affiliations:** ^1^ Department of Chemical and Biomolecular Engineering University of Maryland College Park MD 20740 USA; ^2^ Department of Materials Science and Engineering University of Maryland College Park MD 20740 USA; ^3^ Maryland Nanocenter University of Maryland College Park MD 20740 USA

**Keywords:** Salt in Salt, Carbonate Electrolyte, Dendrite-Free, Inorganic Interphase, Lithium Metal Batteries

## Abstract

The instability of carbonate electrolyte with metallic Li greatly limits its application in high‐voltage Li metal batteries. Here, a “salt‐in‐salt” strategy is applied to boost the LiNO_3_ solubility in the carbonate electrolyte with Mg(TFSI)_2_ carrier, which enables the inorganic‐rich solid electrolyte interphase (SEI) for excellent Li metal anode performance and also maintains the cathode stability. In the designed electrolyte, both NO_3_^−^ and PF_6_^−^ anions participate in the Li^+^‐solvent complexes, thus promoting the formation of inorganic‐rich SEI. Our designed electrolyte has achieved a superior Li CE of 99.7 %, enabling the high‐loading NCM811||Li (4.5 mAh cm^−2^) full cell with N/P ratio of 1.92 to achieve 84.6 % capacity retention after 200 cycles. The enhancement of LiNO_3_ solubility by divalent salts is universal, which will also inspire the electrolyte design for other metal batteries.

## Graphical abstract

A universal salt‐in‐salt strategy is proposed to boost LiNO_3_ solubility in carbonate electrolytes using multivalent salt carrier. The NO_3_^−^‐containing anion complexes and multivalent cations as interphase adsorbing components promote the anion‐derived SEI formation. The designed electrolyte presents a superior Li CE of 99.7 % and enables the NMC811 full cells with N/P ratio of 1.92 to achieve 84.6 % capacity retention after 200 cycles.

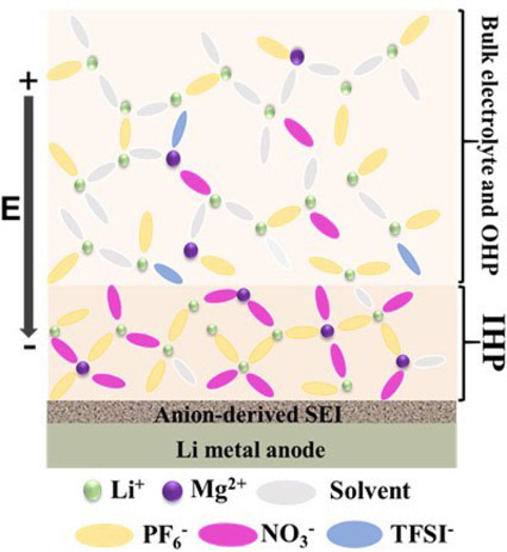

## Introduction

Lithium metal batteries (LMBs) using high‐voltage (>4.3 V) lithium transition metal oxide cathodes such as LiNi_0.80_Co_0.15_Al_0.05_O_2_ (NCA) and LiNi_0.8_Mn_0.1_Co_0.1_O_2_ (NMC811) are the most promising energy storage systems due to the high energy density.[^1^, ^2^, ^3^, ^4^, ^5^, ^6^] However, the dendrite growth and low Coulombic efficiency (CE) of Li metal anodes, cracking evolution and the serious side reactions between electrolytes and the Ni‐rich cathodes under high voltage greatly reduce the cycle life.[^7^, ^8^, ^9^] Due to the highly reductive nature of metallic Li, all the organic solvents and salt anions will be reduced on the Li metal surface, forming solid electrolyte interphase (SEI).[^10^, ^11^, ^12^, ^13^] For commercial carbonate electrolytes, an organic–inorganic SEI is always formed due to the simultaneous reduction of solvent and anions. It cannot effectively suppress Li dendrite growth because the organic–inorganic SEI is strongly bonded to Li and cannot accommodate large volume change of Li, as demonstrated by a low CE of <90 % for Li plating/stripping cycles. Since LiF‐rich interphase has low bonding energy to Li and lithium transition metal oxide cathode, it can accommodate the large volume change during cycling. In addition, LiF has a low solubility in electrolytes and high anodic stability to support high‐voltage cathode chemistries, LiF‐rich interphase is preferred for high‐energy Li||NMC811 cells. To form LiF‐rich interphase on both anode and cathode, the complexation property of electrolyte solvation needs to be regulated.

When lithium salts are dissolved into the solvents, Li^+^‐solvent complexes are formed. The chemical species in the primary Li^+^‐solvent coordination shell are believed to be preferentially reduced and dominate the interfacial chemistry.[^14^, ^15^] Typically, the solvent reduction by metallic Li will form organic–inorganic components while carbon‐free inorganic anion reduction by metallic Li only forms inorganic components. Therefore, an inorganic SEI will be formed if the inorganic anions have a higher reduction potential and the amount of them is larger than that of the solvent in the first solvation sheath. With a lower reduction potential than carbonate solvents, ether solvents can decrease the organic content in SEI by suppressing solvent reduction but have the inherent weaknesses of low anodic stability. To enhance the anodic stability to support high‐voltage cathode as well as achieve high Li plating/stripping CE, the bulk or local salt concentration in ether electrolytes has been increased to form anion‐derived inorganic‐rich interphase on both Li anode and on high‐voltage cathodes, which boosts the Li CE to 99.5 % and enable the LMBs with stable cycling performance.[^16^, ^17^, ^18^, ^19^] Attentively, using fluorinated ether solvent can also form LiF‐rich SEI on Li and robust CEI on the cathode, achieving a long‐cycling life of Li||NMC811 cells.^[20]^ However, Li CE in the best ether electrolyte is still much lower than graphite anode (99.98 %), and the thermal and anodic stability of ether electrolytes still needs further improvement.

The carbonate electrolytes usually promote the formation of organic‐rich SEI on Li metal surface due to its relatively high reduction potential. However, their high stability in anti‐oxidation and against thermal runaway is still quite attractive for high‐voltage LMBs, especially with fluorinated carbonate electrolytes. The inorganic‐rich SEI can still be formed in carbonate electrolytes by fluorinated carbonate electrolytes,[^21^, ^22^] highly concentrated electrolytes (HCEs) or localized HCEs,[^23^, ^24^] or by adding inorganic salts that have a high reduction potential than carbonate solvents.[^25^, ^26^] Lithium nitrate (LiNO_3_) has a higher reduction potential than LiPF_6_ and LiFSI, and can participate in the Li^+^ solvation sheath to form inorganic SEI on Li metal anode.^[27]^ Besides, the nitrate anion (NO_3_^−^) has a lower activation energy than other types of anions, which will be preferentially adsorbed in the inner Helmholtz plane (IHP) with the closer proximity to Li metal surface[^28^, ^29^, ^30^] and thus being firstly reduced to form the interphase for protecting the metallic Li. In addition, with three oxygen atoms carrying a −2/3 charge and one nitrogen atom carrying a +1 charge, the NO_3_^−^ with resonance structures can easily react with metallic Li due to its strong oxidizing ability. This will promote inorganic Li_2_O, LiN_*x*_O_*y*_ and Li_3_N species in the resulted SEI, which can improve the stiffness and ionic conductivity of the electrode interphase.[^31^, ^32^] However, the rather low solubility of LiNO_3_ in carbonate solvents has long restrained its application. To enhance the solubility of LiNO_3_ in carbonate electrolytes_,_ various solvents with a high solubility to LiNO_3_ were used as carriers to form solvent‐in‐salt solution additives. Dimethyl sulfoxide,^[32]^ γ‐Butyrolactone,^[33]^ tris(pentafluorophenyl)borane,^[34]^ and sulfolane^[35]^ carriers have largely increased the LiNO_3_ solubility in carbonate electrolytes, successfully leading to N‐rich inorganic SEI on Li. However, the solvents in these designed solvent‐in‐salt additives also participate the Li^+^ solvation structure, forming organic SEI due to reduction reaction and limiting the Li CE to 99 % especially at a practical capacity loading of>3 mAh cm^−2^.[^36^, ^37^, ^38^] To avoid the side impact of solvent carriers, salt‐in‐salt additive was also developed into electrolytes. For example, CuF_2_‐in‐LiNO_3_ additive was used for ester electrolytes.^[39]^ However, the Cu‐cation reduction also affects the Li plating/stripping efficiency to a value around only 98 %. Exploring the multifunctional salt‐in‐LiNO_3_ additives has not been realized to simultaneously enhance LiNO_3_ solubility in carbonate electrolytes and also promote formation of inorganic SEI for superior Li CE (>99.5 % at>3 mAh cm^−2^), which is highly expected for the practical application of LMBs.

In this contribution, multivalent salts are applied to enhance the LiNO_3_ solubility in the carbonate electrolyte and multivalent cations also regulate Li^+^ solvation structure to reinforce anion absorption as well as reduction on the Li surface. Three multivalent salts with weakly coordinating anions (magnesium bis(trifluoromethanesulfonyl)imide (Mg(TFSI)_2_), zinc bis(trifluoromethanesulfonyl)imide (Zn(TFSI)_2_) and aluminum trifluoromethanesulfonate (Al(OTf)_3_)) have been used as carriers to enhance the LiNO_3_ solubility in the FEC‐EMC based carbonate electrolyte. In the designed electrolytes, NO_3_^−^ successfully participates in the primary Li^+^ solvation sheath and restrains the solvent molecular inside. Meanwhile, the electrostatic interaction between anions and multivalent cations also promotes more NO_3_^−^ and PF_6_^−^ anions adsorption in the IHP for the inorganic‐rich SEI formation. The 1.0 M LiPF_6_‐0.125 M LiNO_3_‐0.025 M Mg(TFSI)_2_ in FEC‐EMC electrolyte has achieved a record‐high CE of 99.7 % at a high capacity of 4.5 mAh cm^−2^, enabling the NCM811||Li full cells with the same areal capacity under a low N/P ratio of 1.92 to achieve a capacity retention of 84.6 % after 200 cycles. The salt‐in‐salt approach presents a sustainable SEI design for aggressive electrochemistry, whose unexplored ion‐solvation structure, electric double layer, and interfacial chemistry also provide exciting insights for the further developments of next‐generation batteries.

## Results and Discussion

**Figure 1:**
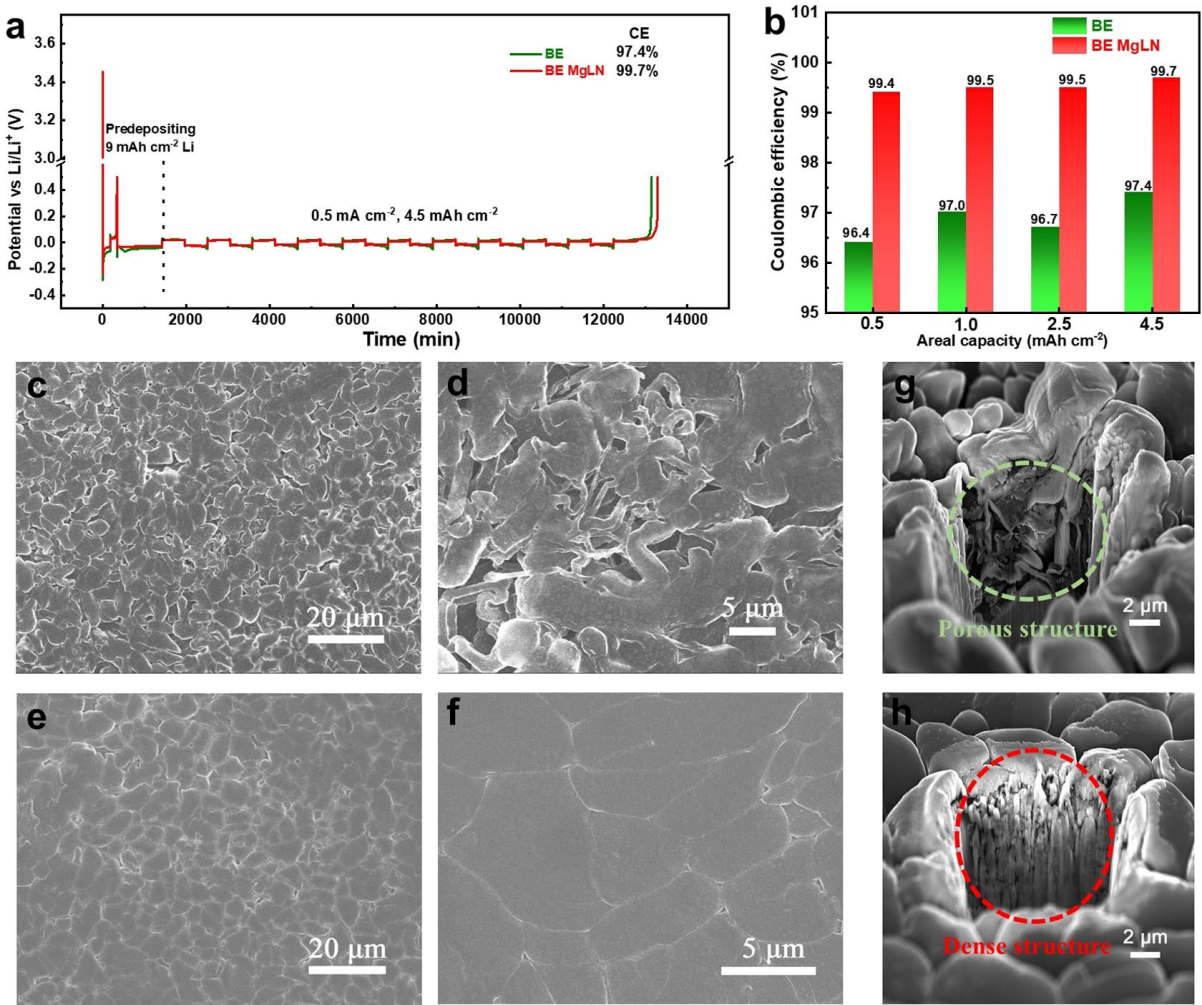
The Li Coulombic efficiency and the electrodeposited Li morphology in different electrolytes. a,b) The typical Li plating/stripping profile and Li CE in BE and BE MgLN electrolytes at 0.5 mA cm^−2^ with different areal capacity under 50 % Li utilization. c–f) Typical SEM images of the plated Li in BE (c,d) and BE MgLN (e,f) electrolytes. Metallic Li is electrochemically deposited on the bare Cu substrate at 0.5 mA cm^−2^ with 4.5 mAh cm^−2^. g,h) The cross‐sectional density comparison of the plated Li metal in BE (g) and BE MgLN (h) electrolytes, of which the crater was sputtered by the Ga^+^ ion beam.

When 25 mmol LiNO_3_ was added into the baseline 1.0 M LiPF_6_ in FEC‐EMC (3 : 7 by vol.) electrolyte (BE), distinct LiNO_3_ precipitation appears at the solution bottom due to the low solubility of LiNO_3_ in carbonate electrolytes. However, the transparent solution is maintained when even 125 mmol LiNO_3_ is added into the same electrolyte using 25 mmol Mg(TFSI)_2_, Zn(TFSI)_2_ or Al(OTf)_3_ as the carrier (Figure S1). The significantly enhanced solubility of LiNO_3_ in carbonate electrolytes is attributed to the solvating ability of multivalent salts. The average CE for Li plating/stripping was evaluated using a 10‐cycle protocol after a preconditioning formation cycle in the Li||Cu cell. As shown in Figure S2, at a current density of 0.5 mA cm^−2^ with a capacity of 0.5 mAh cm^−2^, the metallic Li CE in BE is only 96.5 %, while LiNO_3_‐reinforced electrolytes carried by multivalent salt (Mg(TFSI)_2_ or Zn(TFSI)_2_ or Al(OTf)_3_) achieve a high Li CEs of>98.8 %. The electrolyte with Mg(TFSI)_2_−LiNO_3_ additive (denoted as BE MgLN) even achieves a record‐high CE of 99.6 %. The Li CE value increases following the order of 96.5 % (BE)<98.8 % (LiPF_6_−LiNO_3_−Zn(TFSI)_2_ in FEC‐EMC, denoted as BE ZnLN)<98.9 % (LiPF_6_−LiNO_3_−Al(OTf)_3_ in FEC‐EMC, denoted as BE AlLN)<99.6 % (LiPF_6_−LiNO_3_−Mg(TFSI)_2_ in FEC‐EMC, denoted as BE MgLN). Among these three kinds of electrolytes, BE MgLN electrolyte shows the highest Li CE and appeals the focus for further investigation. The Li CEs in the BE and BE MgLN electrolytes were also evaluated and compared at different capacities. As shown in Figures 1a and 1b, with the increasing Li plating/stripping capacity from 0.5 to 4.5 mAh cm^−2^ at the fixed current of 0.5 mA cm^−2^ and Li utilization of 50 % (Li utilization=cycling capacity/pre‐deposited capacity), the Li CEs in BE show a fluctuation between 96.4 % and 97.4 %, while the value in BE MgLN increases from 99.4 % at 0.5 mAh cm^−2^ to 99.7 % at 4.5 mAh cm^−2^. The high Li plating/stripping CE of 99.7 % at a high capacity of 4.5 mAh cm^−2^ is one of the highest values reported in all carbonate and ether electrolytes (Table S1). Meanwhile, at 1.0 mA cm^−2^ with 1.0 mAh cm^−2^, the CE can also achieve a high value of 99.6 % with 50 % Li utilization (Figure S3).

The typical morphology of Li metal was characterized using scanning electron microscopy (SEM) after 4.5 mAh cm^−2^ Li metal electrodeposited on Cu substrate in both electrolytes, which are displayed in Figures 1c–f. The deposited Li in the BE has nodule‐like (dendrite) morphology and is loosely aggregated together (Figure 1c,d). The needle‐like dendrites with the porous structure can be clearly observed at the higher magnification scale, which reveals the unsteady Li deposition in BE. In contrast, largely bulk Li tightly connected with each other is found in the BE MgLN electrolyte (Figure 1e,f). The chunky Li particles are growing in a planar direction with a much denser structure, which proves the homogeneous Li plating behavior in BE MgLN. The Li metal SEI and protection mechanism will be discussed in the interphase component analysis later. Moreover, the cross‐sectional morphology of the deposited Li was also observed by etching Li metal using the Ga^+^ ion beam sputtering (5 μm×5 μm area). As shown in the SEM images in Figure S4 and Figure 1g, the Li deposited in BE is loosely stacked with significant void propagating throughout the whole film. Therefore in BE, the Li dendrite growth with a porous structure leads to the accumulated SEI with a large contact area between deposited Li and electrolyte, thus resulting in the lower CE along the cycling. However, a much dense packed Li metal is observed in BE MgLN electrolyte (Figure 1h), which is in good agreement with the top‐view morphology (Figure 1f) and the high CE (Figure 1b). Similar morphology difference is also found on the 2 mAh cm^−2^ Li, as shown in Figure S5. The sharp differences of Li CE and Li morphology in those two electrolytes demonstrate that the advanced SEI formed in BE MgLN electrolyte greatly suppresses the Li dendrite growth.

**Figure 2:**
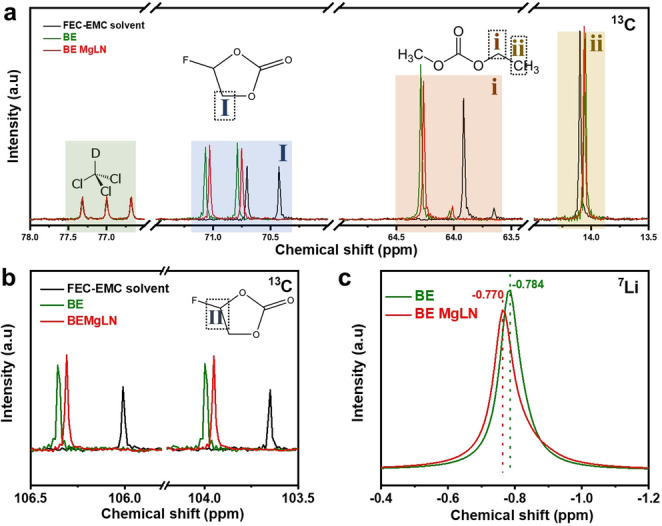
The solvation structure characterization in BE and BE MgLN electrolytes. a,b) The ^13^C nuclear magnetic resonance (NMR) spectra of pure FEC‐EMC solvent, BE and BE MgLN electrolytes. c) ^7^Li NMR spectra in BE and BE MgLN electrolytes.

The coordination‐solvation structures of electrolytes with or without salt‐in‐salt additive were characterized by Nuclear Magnetic Resonance (NMR) spectroscopy. The deuterated chloroform (CDCl_3_) is applied as the internal reference solvent for the chemical shift, of which the carbon has triplet peaks around 77 ppm with the same intensity due to deuterium coupling. As displayed in Figures 2a,b and Figures S6,S7, the 13 C chemical shift in the BE exhibits a downfield shift compared with the pure FEC‐EMC solvent. It proves the reduced interaction between Li^+^ and ester molecules in the solvation sheath and thus the electron density around the carbon nucleus decreases due to the deshielding effect. However, with Mg(TFSI)_2_−LiNO_3_ additive, all chemical shifts of ^13^C in the carbonate solvents gradually shift upfield, indicating the higher electron density around the nucleus. It demonstrates that more NO_3_^−^ and TFSI^−^ anions have participated in the solvation structure of Li^+^ and replaced EMC and FEC molecular, thus weakening their dipolar interaction and resulting in the shielding effect. Meanwhile, the ^7^Li spectra also confirm the changes of solvation chemistry. The ^7^Li chemical shift of BE MgLN (−0.770 ppm) is slightly larger than that of BE electrolyte (−0.783 ppm) (Figure 2c), demonstrating the deshielding effect of the Li^+^ solvation sheath. Mg^2+^ cations are also involved in solvation structures due to the higher charge and strong electron‐withdrawing properties, thus reducing the solvent molecule number surrounding the Li^+^ as well as the electron density. Based on the NMR characterizations, NO_3_^−^ anions in BE MgLN electrolyte have participated in the primary Li^+^ solvation sheath and Mg^**2+**^ cations on Li SEI surface also attract NO_3_^−^ and PF_6_^−^ adsorption. The involvement of both the Mg(TFSI)_2_ and LiNO_3_ in the coordination‐solvation structure of the BE MgLN electrolyte promotes the reductions of PF_6_^−^ and NO_3_^−^ anions to form/heal inorganic SEI. The reduction potential of NO_3_^−^ is also evaluated using cyclic voltammetry (CV) in Li||Cu cell at a scanning rate of 0.1 mV s^−1^. As shown in Figure S8, the BE MgLN shows a distinct reduction peak around 1.5 V during the cathodic scan, which can be attributed to the reductive decomposition of NO_3_^−^ for the inner SEI on the Li metal surface. Since polyanionic structures reduce the electrostatic force of NO_3_^−^ with Li^+^, the solubility of LiNO_3_ in carbonate electrolytes is enhanced. The involvement of both the Mg(TFSI)_2_ and LiNO_3_ in the coordination‐solvation structure of the BE MgLN electrolyte also reduces the desolvation energy, which will increase the charge‐transfer reaction rate. The exchange current density (i_o_) of Li plating/stripping was measured in symmetrical Li||Li cells using linear sweep voltammetry (LSV) between ‐0.2 to 0.2 V at a scan rate of 1.0 mV s^−1^ (Figure S9). The i_o_ in the BE MgLN (46 μA cm^−2^) increased by around 35 % more than that in the BE electrolyte (34 μA cm^−2^), which also validates the better kinetic properties for homogeneous Li^+^ transport in our designed electrolyte.

**Figure 3:**
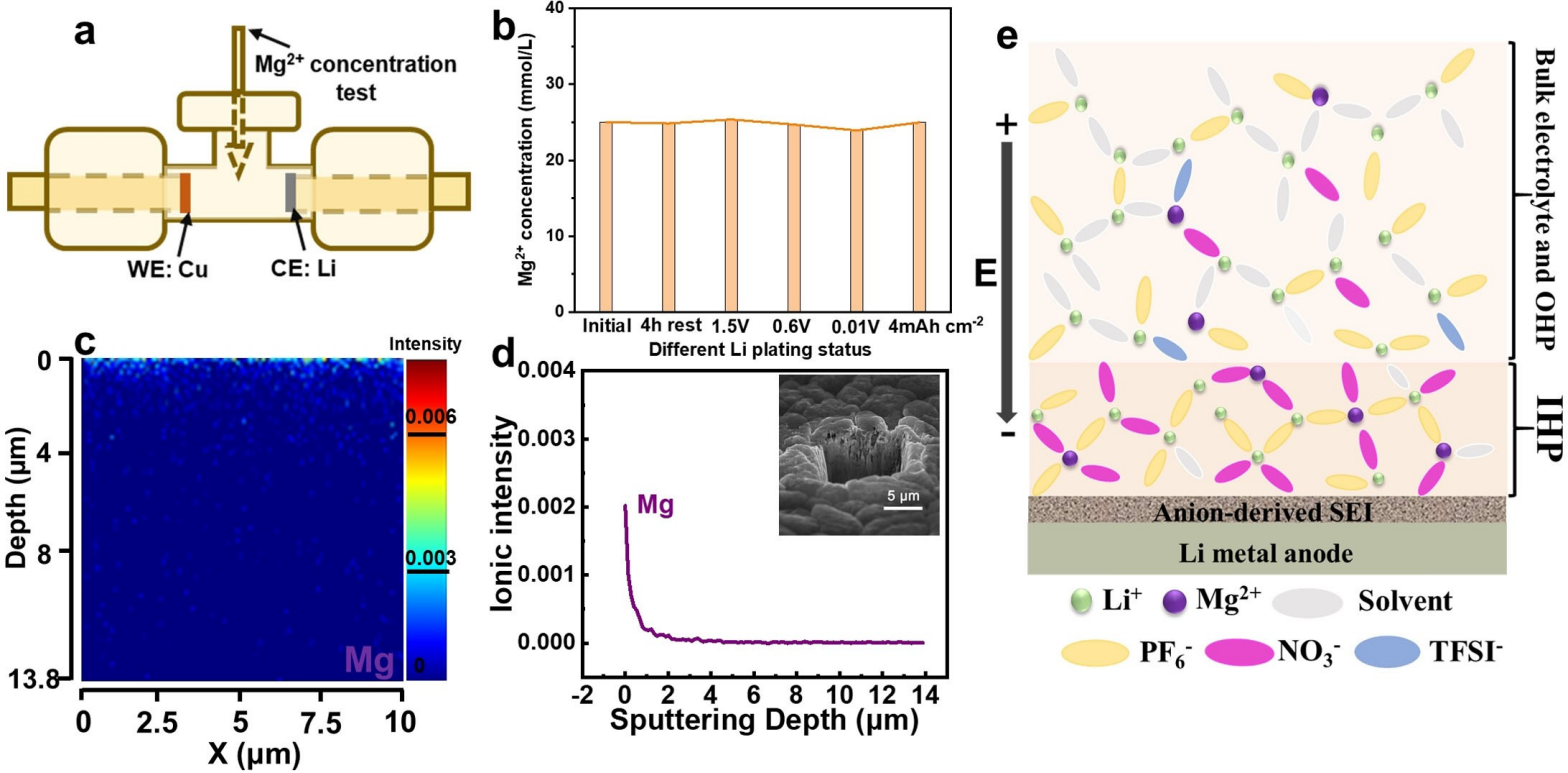
The magnesium ion degradation and schematic diagram of interphase along cycling in BE MgLN. a) The schematic diagram of the two‐electrode cell for Mg^2+^ concentration test by ICP, b) the Mg^2+^ concentration variation of BE MgLN under the decreasing potential of Li||Cu cell, c,d) TOF‐SIMS analysis for the Mg element and its distribution of the cross‐sectional surface under Ga^+^ sputtering (inset with the crater morphology in figure d). e) The schematic description of the role of multivalence ion in the formation of anion‐derived SEI on Li metal anode surface in the designed BE MgLN electrolyte.

Since the reduction potential of 25 mmol Mg^2+^ cations is 0.63 V vs. Li (‐2.41 V vs. SHE), thermodynamically, Mg^2+^ may be reduced into metallic Mg on the Li surface if Mg^2+^ can diffuse through SEI. However, the dynamic diffusion of Mg^2+^ through SEI is extremely slow,[^40^, ^41^] preventing its reduction on Li metal covered with SEI. The possibility for Mg^2+^ reduction on Li was investigated by inductively coupled plasma (ICP), which monitors the Mg^2+^ concentration during the Li plating process in a home‐designed T‐type Li||Cu cell with flooded BE MgLN electrolyte Figure 3a). As shown in Figure 3b, the Mg^2+^ amount in electrolytes does not decrease under the whole rest or different Li plating statue of the Li||Cu cell. Even after 4.0 mAh cm^−2^ Li are plated on Cu, the Mg^2+^ concentration in the BE MgLN is still comparable to its initial value. In addition, to measure Mg^2+^ concentration in BE MgLN electrolyte, Mg concentration on Li metal was also monitored using time‐of‐flight secondary ion mass spectroscopy (ToF‐SIMS). As shown in Figures 3c,d, the 4 mAh cm^−2^ electrodeposited Li metal is carved by the Ga^+^ ion beam over a 10 μm×10 μm area for the element spectroscopy. Only a very limited amount of Mg has been detected on the surface layer, and its signal has decreased rapidly in the whole 14 μm sputtering depth, proving that no Mg^2+^ ions have been deposited inside the Li metal but only on the SEI surface. Meanwhile, highly sensitive X‐ray photoelectron spectroscopy (XPS) is employed to analyze the chemical composition on SEI surface. As displayed in Figure S10a, the Mg signal in SEI is always quite weak during the different Li plating capacity. The main element components in the SEI are Li, C, F and O. The Mg 1s spectra all remain the same position of Mg^2+^ around 1304 eV (Figure S10b). Theoretically, there should also be no Mg^2+^ reduction under a high voltage 1.2 V as well and it further proves that the surface Mg signal is mainly from Mg(TFSI)_2_ salt precipitation. All these characterizations demonstrate that Mg^2+^ ions reduction in BE MgLN has been effectively restrained since the Li metal SEI blocks Mg^2+^ diffusion onto the Li surface,[^42^, ^43^] and our recent work also demonstrated that Mg^2+^ can only achieve efficient deposition on SEI‐free substrate.^[43]^

Based on the characterizations above, a conceptual solvation structure of BE MgLN electrolyte near to Li anode surface was proposed (Figure 3e). During the Li plating process, the Li^+^‐solvent coordination structure with NO_3_^−^ and PF_6_^−^ anions will transport from the bulk electrolyte area to the IHP on the Li metal surface. Due to the nanoscale size of IHP, solvated cations will undergo the desolvation process first and ions including Li^+^, Mg^2+^, NO_3_^−^, PF_6_^−^, TFSI^−^ and FEC, EMC molecules are actually in a dynamically competitive state for adsorption in the IHP. Under the electrostatic field of the Li electrodeposition process, a trace amount of divalent Mg^2+^ is also speculated to adsorb in the IHP due to its higher charge. Mg^2+^ cations rather only act more as the interphase adsorbing component in the electrolyte, which will promote more NO_3_^−^ and PF_6_^−^ anions in the IHP due to the stronger electrostatic interaction between anions and divalent cations and push organic solvent away from the interface. It is proven that the adsorbed species in IHP, closest to the Li metal surface in the electrolyte, are very critical in achieving a stable SEI layer. As a result, the preferential decomposition NO_3_^−^ and PF_6_^−^ anions forms the fluoride/nitride mixed SEI on the Li metal surface, which simultaneously improves the electrochemical stability and Li‐ion conductivity of the anion‐derived SEI.

**Figure 4:**
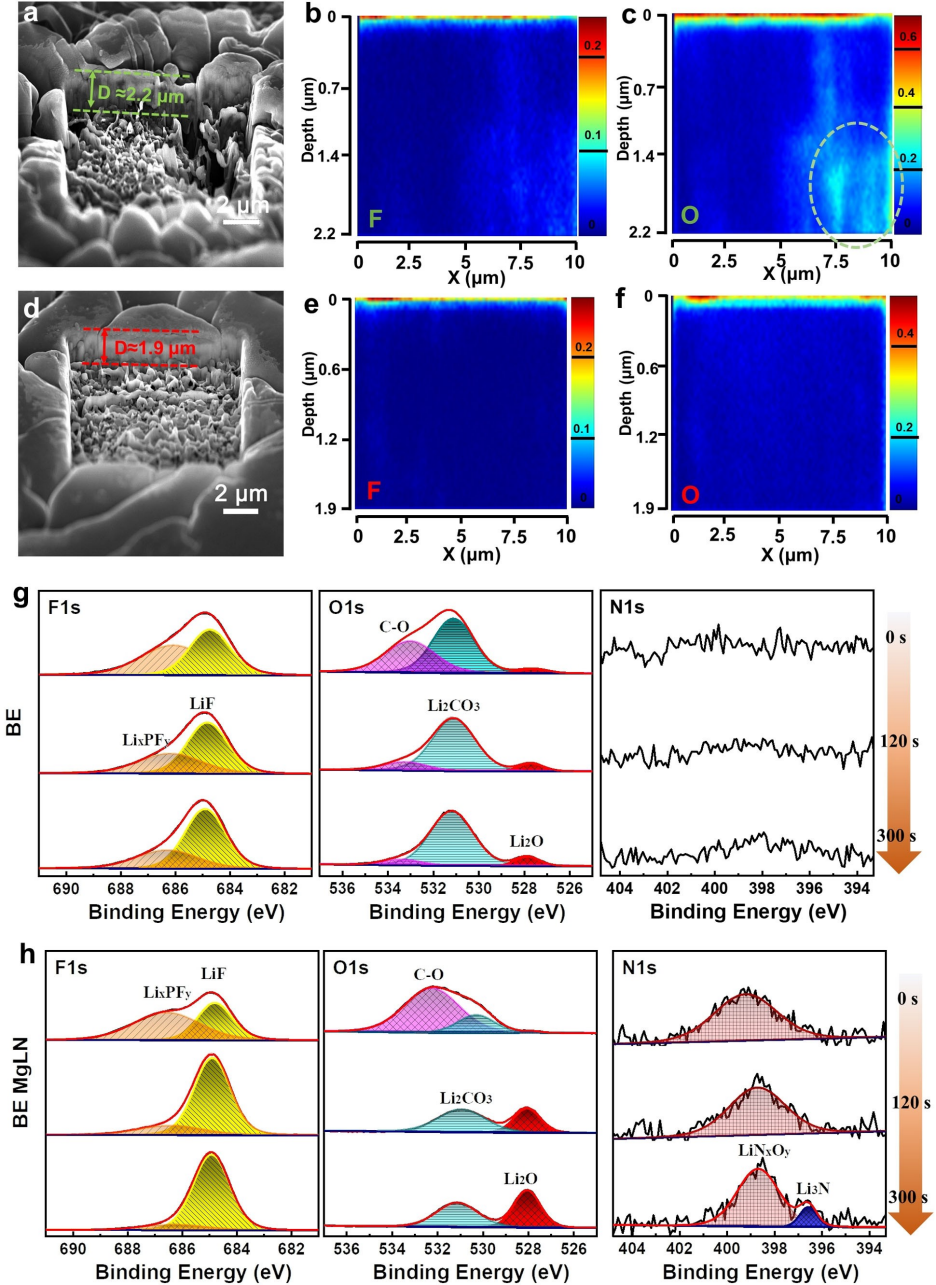
SEI characterizations in different electrolytes. a–f) The elemental F and O distributions in the sputtered cross‐section crater of the electrochemically deposited Li surface in BE (a–c) and BE MgLN (d–f) by Tof‐SIMS using Ga^+^ beam over a 10 μm×10 μm area. g,h) The typical O1s, N1s, and F1s spectra of the SEI layer formed in BE (g) and BE MgLN (h) electrolytes by the in‐depth XPS measurement.

The SEI compositions formed in the BE and the BE MgLN were characterized by ToF‐SIMS with continuous Ga^+^ sputtering. As shown in Figures 4a and 4d, the crater edge of the Li metal anode in BE and BE MgLN presents a sputtering depth of around 2 μm with an etching area of 10 μm×10 μm. The elements have been captured and quantitatively analyzed over the entire three‐dimensional region with the cross‐sectional element distribution shown in Figures 4b,c, and Figures 4e,f. In the negative mode, obvious F and O signals were found on the Li metal surface after cycling in both electrolytes, which reveals the formed SEI region. According to the collected signal points, the F and O elements are taking a volume of 0.92 % and 17.47 % respectively for the electrodeposited Li in BE (Figure S11). Especially the O signal heterogeneously aggregates over the entire longitudinal section, which reveals the side reaction of metallic Li to carbonate solvents. The undesired microstructure with whisker dendrite and more tortuosities can easily produce the SEI with a larger area and may result in more inactive Li metal during the stripping process. In sharp contrast, a distinct distribution of F and O signals only aggregate on the top of the Li metal surface in BE MgLN, which takes a volumetric percentage of 1.48 % and 4.11 % separately (Figure S12). This is because the chunky Li with a dense structure and less tortuosity have intimate connections to maintain its bulk integrity, resulting in the reduced side reaction between the active metallic Li and electrolyte. It also further verifies that a highly stable SEI is maintained on the Li metal surface in our designed electrolyte, thus ensuring the columnar Li microstructure with minimum tortuosity as well as its outstanding CE.

X‐ray photoelectron spectroscopy (XPS) was employed to unveil the SEI compositions on the Li metal surface after cycling in BE and BE MgLN electrolytes. The SEI on the Li metal anode in BE mainly consists of organic compounds (C−O, C−C peaks) with a small amount of inorganic compounds such as Li_*x*_PF_y_, LiF, and Li_2_CO_3_ (Figure 4g and Figure S13a). All the organic and inorganic peaks are also well‐maintained without enormous differences after etching, which proves that the SEI in BE is enriched with organic–inorganic mixed components from the top to the bottom. In comparison, a clear peak of LiN_*x*_O_y_ appears in the SEI formed in the BE MgLN electrolyte due to the decomposition of LiNO_3_ (Figure 4h). Under the whole 5 min sputtering, stronger LiF and Li_2_O peaks are also found in the derived SEI as well as the new emergence of the Li_3_N peak. Meanwhile, C−O and C−C peaks present a distinct attenuation in the C1s spectra, indicating much fewer organic compounds in the inner SEI part closer to metallic Li (Figure S13b). The atomic ratio of the detailed compositions is also compared in Figure S14. For the SEI in BE, the C atomic percentage, representative of the organic species, decreases slightly after 5 minutes of sputtering but still maintained a high percentage of 22.4 %, indicating organic components are enriched in the entire SEI. By contrast, the C ratio has been sharply decreased to only 8.9 % after the same etching, while the total amount of the Li, O and F elements reaches as high as 88.3 %, validating that a highly inorganic‐rich interphase has been formed in the designed electrolyte.

The formation of inorganic‐rich SEI with LiF, Li_2_O, and nitride compounds on the Li metal surface in BE MgLN electrolytes is attributed to the reduction of PF_6_^−^ and NO_3_^−^, which are well adsorbed in the IHP due to the electrostatic interaction of divalent Mg^2+^. The nitrides including Li_3_N and LiN_*x*_O_y_ are excellent ionic conductors for lithium ions, which can improve the ionic transport of the derived SEI. Meanwhile, LiF and Li_2_O typically exhibit lithiophobicity properties (high interface energy with metallic Li) and high Young's modulus, thus can suppress dendrite growth.[^44^, ^45^] Owning to the high interfacial energy with Li metal, improved ion‐transport capability as well as high mechanical properties, the inorganic‐rich SEI can promote the Li lateral growth and suppress dendrite from penetrating the interface, thus contributing to the outstanding electrochemical performance of LMBs.

**Figure 5:**
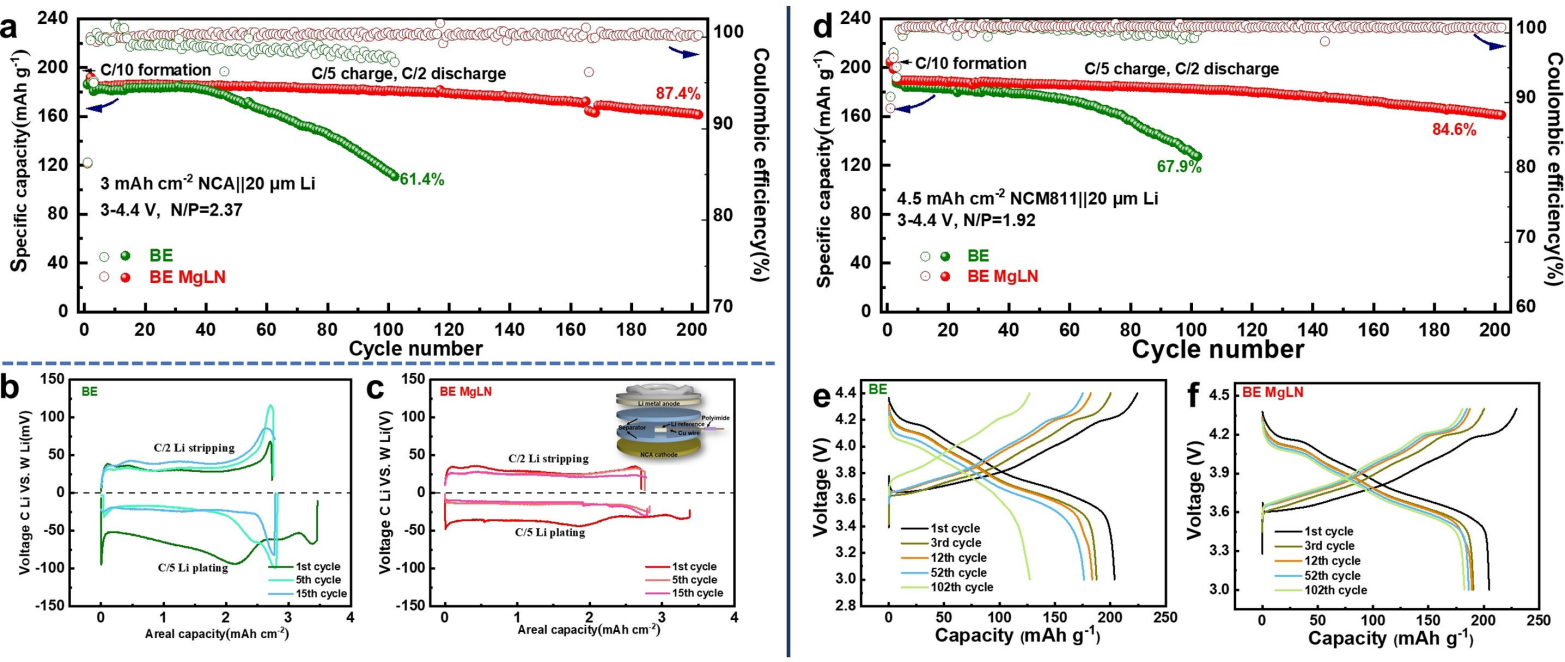
Electrochemical performances of LMBs in BE and BE MgLN. a) Cycling performances of Li||NCA full cell with a capacity loading of 3 mAh cm^−2^. b,c) Voltage profile of the counter Li anode to reference Li electrode in 1^st^, 5^th^ and 15^th^ cycles of the 3‐electrode Li||NCA cell (cell configuration setup inset in figure c). d) Cycling performances of Li||NCM811 full cell with a capacity loading of 4.5 mAh cm^−2^ and e,f) the corresponding charging/discharging profiles in various cycles.

Since carbonate electrolytes have high anti‐oxidation stability and can support high voltage cathode, the full cells using high energy NCA (LiNi_0.80_Co_0.15_Al_0.05_O_2_) and NMC811 (LiNi_0.8_Mn_0.1_Co_0.1_O_2_) cathodes and Li anodes were evaluated in different electrolytes at a high charging cutoff voltage of 4.4 V. For Li||NCA cells, two formation cycles at C/10 were firstly conducted before the long‐term cycling at a higher rate between the cycling range of 2.7–4.4 V. The electrochemical performance of 20 μm Li||NCA full cells with an area capacity of 3 mAh cm^−2^ is displayed in Figure 5a and the N/P ratio is 2.37 (1.37 fold Li excess) when considering the Li amounts in both anode and cathode. The NCA full cells in both electrolytes deliver a specific capacity of around 186 mAh g^−1^ in the first cycle. For the cell with BE, its capacity displays a continuous decay after 40 stable cycles and shows a 61.4 % retention after 100 cycles. In contrast, long‐term cycling stability is found for the BE MgLN, maintaining an outstanding capacity retention of 87.4 % after 200 cycles. The cell impedances at different cycles at the discharging state of 3 V are measured using electrochemical impedance spectroscopy (EIS, Figure S15). The semicircle at high frequencies of Nyquist plots can be attributed to Li^+^ diffusion through the electrode interphase and the radius value can be assigned to the interphase resistance (R_SEI_). In BE MgLN, the R_SEI_ exhibits a similar value around 9 Ω at both the 5^th^ and 15^th^ cycles with almost no increase. However, the R_SEI_ has increased from 20 Ω at the 5^th^ to 34 Ω at the 15^th^ cycle in BE, which is mainly due to the unstable electrode interphase with an accumulated thickness along cycling.

The charge/discharge profile of NCA cathode and Li anodes (Figure S16) was monitored separately by using a three‐electrode full cell (the cell structure inset in Figure 5c) with Li metal coated on Cu as the reference electrode. The enlarged view of the Li plating/stripping overpotential during the entire cycling process is plotted in Figures 5b, c. A much smoother voltage plateau as well as smaller polarizations can be found in BE MgLN, which reveals that a more stable SEI with improved kinetics has been constructed for the uniform Li deposition/dissolution. However, irregular voltage fluctuations with the larger hysteresis peak happen at the end of each Li plating/stripping process in BE electrolyte. It is mainly because the strong bonding between the organic‐rich SEI in BE with metallic Li cannot bear the huge volume expansion during cycling, thus resulting in the repeated break/reformation of the SEI and the accompanied nucleation of newly mossy‐like dendrite Li or pitting(Li dissolution from the bulk surface) with larger cell polarization.[^46^, ^47^] The different Li overpotentials affect the delivered potential of the full cell as well as the capacity. As shown in Figures S17 and S18, due to the large overpotential of Li metal anode with unstable interphase in BE, the potential gap of NCA cathode to counter Li anode and reference Li electrode is always larger than that in BE MgLN, which also results in the less stable working potential along the cycles.

The Li||NMC811full cells with a higher capacity of 4.5 mAh cm^−2^ and a lower N/P ratio of 1.92 (0.92 fold Li excess) were also evaluated in both electrolytes. As shown in Figure 5d, the BE delivers a poor capacity retention of 67.9 % after 100 cycles, while the BE MgLN still maintains an excellent capacity retention of 84.6 % after 200 cycles. Figures 5e and 5 f show the voltage‐capacity evolution profiles in BE and BE MgLN during the initial 100 cycles. In specific, the cell with BE delivers a discharging capacity of 176.4 and 127.5 mAh g^−1^ after 50 and 100 cycles, respectively. In contrast, a much‐improved cycling performance has been achieved using the BE MgLN, of which the discharging capacity still reaches a high value of 186.5 after 50 cycles and 182.4 mAh g^−1^ after 100 cycles. Such a huge difference further demonstrates the importance of Mg(TFSI)_2_−LiNO_3_ additive in higher capacity retention and longer lifespan of rechargeable LMBs. When compared to the other reported battery results in advanced electrolytes (Table S2), our designed BE MgLN also exhibits one of the best performances for high‐voltage LMBs under the high areal loading and low N/P ratio.

## Conclusion

In summary, a universal salt‐in‐salt strategy has been developed to construct a stable inorganic‐rich SEI of Li metal anode in the carbonate electrolyte. Less‐soluble LiNO_3_ was successfully introduced into the FEC‐EMC based carbonate electrolyte using Mg(TFSI)_2_ as a carrier. NO_3_^−^ anions have participated in the Li^+^ solvation structure of carbonate electrolytes while the multivalent Mg^2+^ cations on the SEI surface attract additional PF_6_^−^ and NO_3_^−^ anions, promoting the formation of inorganic SEI enriched in nitride/fluoride components. Due to the difficult diffusion through the Li metal SEI, Mg^2+^ cations are well maintained in the electrolyte. Such a stable interphase with high interfacial energy and high ionic conductivity effectively regulates the uniform Li deposition and protects active Li from electrolyte corrosion. As a result, the designed LiPF_6_−LiNO_3_−Mg(TFSI)_2_ in FEC‐EMC electrolyte has achieved an outstanding CE of 99.7 % at a high capacity of 4.5 mAh cm^−2^. The 4.5 mAh cm^−2^ NCM811||Li full cell with N/P of 1.92 maintains 84.6 % capacity retention after 200 cycles. This work sheds light on the advanced electrolyte design, which will inspire further research about LMBs as well as sodium and potassium metal batteries.

## Conflict of interest

The authors declare no conflict of interest.

1

## Supporting information

As a service to our authors and readers, this journal provides supporting information supplied by the authors. Such materials are peer reviewed and may be re‐organized for online delivery, but are not copy‐edited or typeset. Technical support issues arising from supporting information (other than missing files) should be addressed to the authors.

Supporting Information

## Data Availability

The data that support the findings of this study are available in the supplementary material of this article.

## References

[1] D. Lin , Y. Liu , Y. Cui , Nat. Nanotechnol. 2017, 12, 194–206. 28265117 10.1038/nnano.2017.16

[2] W. Liu , P. Oh , X. Liu , M. J. Lee , W. Cho , S. Chae , Y. Kim , J. Cho , Angew. Chem. Int. Ed. 2015, 54, 4440–4457; 10.1002/anie.201409262 25801735

[3] M. D. Tikekar , S. Choudhury , Z. Tu , L. A. Archer , Nat. Energy 2016, 1, 16114.

[4] X. B. Cheng , R. Zhang , C. Z. Zhao , Q. Zhang , Chem. Rev. 2017, 117, 10403–10473. 28753298 10.1021/acs.chemrev.7b00115

[5] J. M. Tarascon , M. Armand , Nature 2001, 414, 359–367. 11713543 10.1038/35104644

[6] J. B. Goodenough , K. S. Park , J. Am. Chem. Soc. 2013, 135, 1167–1176. 23294028 10.1021/ja3091438

[7] W. Xu , J. Wang , F. Ding , X. Chen , E. Nasybulin , Y. Zhang , J.-G. Zhang , Energy Environ. Sci. 2014, 7, 513–537.

[8] B. Thirumalraj , T. T. Hagos , C. J. Huang , M. A. Teshager , J. H. Cheng , W. N. Su , B. J. Hwang , J. Am. Chem. Soc. 2019, 141, 18612–18623. 31642662 10.1021/jacs.9b10195

[9] A. Wang , S. Kadam , H. Li , S. Shi , Y. Qi , npj Comput. Mater. 2018, 4, 15.

[10] E. Peled , S. Menkin , J. Electrochem. Soc. 2017, 164, A1703–A1719.

[11] E. Peled , J. Electrochem. Soc. 1979, 126, 2047–2051.

[12] J. E. Harlow , X. Ma , J. Li , E. Logan , Y. Liu , N. Zhang , L. Ma , S. L. Glazier , M. M. E. Cormier , M. Genovese , S. Buteau , A. Cameron , J. E. Stark , J. R. Dahn , J. Electrochem. Soc. 2019, 166, A3031–A3044.

[13] G. Xu , X. Shangguan , S. Dong , X. Zhou , G. Cui , Angew. Chem. Int. Ed. 2020, 59, 3400–3415; 10.1002/anie.201906494 31332946

[14] X. Chen , Q. Zhang , Acc. Chem. Res. 2020, 53, 1992–2002. 32883067 10.1021/acs.accounts.0c00412

[15] X. Ren , P. Gao , L. Zou , S. Jiao , X. Cao , X. Zhang , H. Jia , M. H. Engelhard , B. E. Matthews , H. Wu , H. Lee , C. Niu , C. Wang , B. W. Arey , J. Xiao , J. Liu , J. G. Zhang , W. Xu , Proc. Natl. Acad. Sci. USA 2020, 117, 28603–28613. 33144505 10.1073/pnas.2010852117 PMC7682554

[16] N. Piao , X. Ji , H. Xu , X. Fan , L. Chen , S. Liu , M. N. Garaga , S. G. Greenbaum , L. Wang , C. Wang , X. He , Adv. Energy Mater. 2020, 10, 1903568.

[17] X. Ren , L. Zou , X. Cao , M. H. Engelhard , W. Liu , S. D. Burton , H. Lee , C. Niu , B. E. Matthews , Z. Zhu , C. Wang , B. W. Arey , J. Xiao , J. Liu , J.-G. Zhang , W. Xu , Joule 2019, 3, 1662–1676.

[18] S. Chen , J. Zheng , L. Yu , X. Ren , M. H. Engelhard , C. Niu , H. Lee , W. Xu , J. Xiao , J. Liu , J.-G. Zhang , Joule 2018, 2, 1548–1558.

[19] J. Qian , W. A. Henderson , W. Xu , P. Bhattacharya , M. Engelhard , O. Borodin , J. G. Zhang , Nat. Commun. 2015, 6, 6362. 25698340 10.1038/ncomms7362 PMC4346622

[20] Z. Yu , H. Wang , X. Kong , W. Huang , Y. Tsao , D. G. Mackanic , K. Wang , X. Wang , W. Huang , S. Choudhury , Y. Zheng , C. V. Amanchukwu , S. T. Hung , Y. Ma , E. G. Lomeli , J. Qin , Y. Cui , Z. Bao , Nat. Energy 2020, 5, 526–533.

[21] L. Suo , W. Xue , M. Gobet , S. G. Greenbaum , C. Wang , Y. Chen , W. Yang , Y. Li , J. Li , Proc. Natl. Acad. Sci. USA 2018, 115, 1156–1161. 29351993 10.1073/pnas.1712895115 PMC5819397

[22] E. Markevich , G. Salitra , F. Chesneau , M. Schmidt , D. Aurbach , ACS Energy Lett. 2017, 2, 1321–1326.

[23] X. Fan , L. Chen , X. Ji , T. Deng , S. Hou , J. Chen , J. Zheng , F. Wang , J. Jiang , K. Xu , C. Wang , Chem 2018, 4, 174–185.

[24] X. Fan , L. Chen , O. Borodin , X. Ji , J. Chen , S. Hou , T. Deng , J. Zheng , C. Yang , S. C. Liou , K. Amine , K. Xu , C. Wang , Nat. Nanotechnol. 2018, 13, 715–722. 30013215 10.1038/s41565-018-0183-2

[25] X. R. Chen , Y. X. Yao , C. Yan , R. Zhang , X. B. Cheng , Q. Zhang , Angew. Chem. Int. Ed. 2020, 59, 7743–7747; 10.1002/anie.202000375 32160379

[26] X. Liang , Z. Wen , Y. Liu , M. Wu , J. Jin , H. Zhang , X. Wu , J. Power Sources 2011, 196, 9839–9843.

[27] S. H. Lee , J.-Y. Hwang , J. Ming , Z. Cao , H. A. Nguyen , H.-G. Jung , J. Kim , Y.-K. Sun , Adv. Energy Mater. 2020, 10, 2000567.

[28] C. Yan , H. R. Li , X. Chen , X. Q. Zhang , X. B. Cheng , R. Xu , J. Q. Huang , Q. Zhang , J. Am. Chem. Soc. 2019, 141, 9422–9429. 31117672 10.1021/jacs.9b05029

[29] L. Cao , D. Li , E. Hu , J. Xu , T. Deng , L. Ma , Y. Wang , X. Q. Yang , C. Wang , J. Am. Chem. Soc. 2020, 142, 21404–21409. 33290658 10.1021/jacs.0c09794

[30] W. Zhang , Q. Wu , J. Huang , L. Fan , Z. Shen , Y. He , Q. Feng , G. Zhu , Y. Lu , Adv. Mater. 2020, 32, 2001740. 10.1002/adma.202001740 32390225

[31] Y. Liu , D. Lin , Y. Li , G. Chen , A. Pei , O. Nix , Y. Li , Y. Cui , Nat. Commun. 2018, 9, 3656. 30194431 10.1038/s41467-018-06077-5 PMC6128910

[32] S. Liu , X. Ji , N. Piao , J. Chen , N. Eidson , J. Xu , P. Wang , L. Chen , J. Zhang , T. Deng , S. Hou , T. Jin , H. Wan , J. Li , J. Tu , C. Wang , Angew. Chem. Int. Ed. 2021, 60, 3661–3671; 10.1002/anie.202012005 33166432

[33] Y. Jie , X. Liu , Z. Lei , S. Wang , Y. Chen , F. Huang , R. Cao , G. Zhang , S. Jiao , Angew. Chem. Int. Ed. 2020, 59, 3505–3510; 10.1002/anie.201914250 31880025

[34] S. Li , W. Zhang , Q. Wu , L. Fan , X. Wang , X. Wang , Z. Shen , Y. He , Y. Lu , Angew. Chem. Int. Ed. 2020, 59, 14935–14941; 10.1002/anie.202004853 32410377

[35] N. Piao , S. Liu , B. Zhang , X. Ji , X. Fan , L. Wang , P.-F. Wang , T. Jin , S.-C. Liou , H. Yang , J. Jiang , K. Xu , M. A. Schroeder , X. He , C. Wang , ACS Energy Lett. 2021, 6, 1839–1848.

[36] P. Albertus , S. Babinec , S. Litzelman , A. Newman , Nat. Energy 2018, 3, 16–21.

[37] J. L. Schaefer , Y. Lu , S. S. Moganty , P. Agarwal , N. Jayaprakash , L. A. Archer , Appl. Nanosci. 2012, 2, 91–109.

[38] J. Liu , Z. Bao , Y. Cui , E. J. Dufek , J. B. Goodenough , P. Khalifah , Q. Li , B. Y. Liaw , P. Liu , A. Manthiram , Y. S. Meng , V. R. Subramanian , M. F. Toney , V. V. Viswanathan , M. S. Whittingham , J. Xiao , W. Xu , J. Yang , X.-Q. Yang , J.-G. Zhang , Nat. Energy 2019, 4, 180–186.

[39] C. Yan , Y. X. Yao , X. Chen , X. B. Cheng , X. Q. Zhang , J. Q. Huang , Q. Zhang , Angew. Chem. Int. Ed. 2018, 57, 14055–14059; 10.1002/anie.201807034 30094909

[40] H. Dong , Y. Liang , O. Tutusaus , R. Mohtadi , Y. Zhang , F. Hao , Y. Yao , Joule 2019, 3, 782–793.

[41] S. B. Son , T. Gao , S. P. Harvey , K. X. Steirer , A. Stokes , A. Norman , C. Wang , A. Cresce , K. Xu , C. Ban , Nat. Chem. 2018, 10, 532–539. 29610460 10.1038/s41557-018-0019-6

[42] H. Dong , O. Tutusaus , Y. Liang , Y. Zhang , Z. Lebens-Higgins , W. Yang , R. Mohtadi , Y. Yao , Nat. Energy 2020, 5, 1043–1050.

[43] S. Hou , X. Ji , K. Gaskell , P.-F. Wang , L. Wang , J. Xu , R. Sun , O. Borodin , C. Wang , Science 2021, 374, 172–178. 34618574 10.1126/science.abg3954

[44] X. Ji , S. Hou , P. Wang , X. He , N. Piao , J. Chen , X. Fan , C. Wang , Adv. Mater. 2020, 32, 2002741. 10.1002/adma.202002741 33035375

[45] S. Liu , X. Ji , J. Yue , S. Hou , P. Wang , C. Cui , J. Chen , B. Shao , J. Li , F. Han , J. Tu , C. Wang , J. Am. Chem. Soc. 2020, 142, 2438–2447. 31927894 10.1021/jacs.9b11750

[46] K. N. Wood , E. Kazyak , A. F. Chadwick , K. H. Chen , J. G. Zhang , K. Thornton , N. P. Dasgupta , ACS Cent. Sci. 2016, 2, 790–801. 27924307 10.1021/acscentsci.6b00260 PMC5126712

[47] K. N. Wood , M. Noked , N. P. Dasgupta , ACS Energy Lett. 2017, 2, 664–672.

