# Supplementary material for: Salt‐in‐Salt Reinforced Carbonate Electrolyte for Li Metal Batteries
Source: Angew Chem Int Ed Engl. 2022 Sep 21;61(43):e202210522. doi: 10.1002/anie.202210522 (PMC9826201; doi:10.1002/anie.202210522)
Supplement: Supplementary file 1 — Supporting Information [file ANIE-61-0-s001.pdf]

Angewandte Chemie
Eine Zeitschrift der Gesellschaft Deutscher Chemiker

Supporting Information

Salt-in-Salt Reinforced Carbonate Electrolyte for Li Metal Batteries

*S. Liu, J. Xia, W. Zhang, H. Wan, J. Zhang, J. Xu, J. Rao, T. Deng, S. Hou, B. Nan, C. Wang**

Experimental Section

Materials. Lithium hexafluorophosphate ($LiPF_6$), ethyl methyl carbonate (EMC) and fluoroethylene carbonate (FEC) are purchased from Gotion Company in America and are all at battery grade. Lithium nitrate ($LiNO_3$, 99.99%) and aluminum trifluoromethanesulfonate ($Al(OTf)_3$, 99.9%) are bought from Sigma-Aldrich. Magnesium bis(trifluoromethanesulfonyl)imide ($Mg(TFSI)_2$, 99.5%), zinc bis(trifluoromethanesulfonyl)imide ($Zn(TFSI)_2$, 99.5%) are purchased from Solvionic. $LiNO_3$, $Mg(TFSI)_2$, $Al(OTf)_3$ were all dried at 200 °C and $Zn(TFSI)_2$ was dried at 120 °C under vacuum for 12 hours before use. All the solvents were dried by preactivated molecular sieves (4Å, Sigma-Aldrich) before use. The $LiNi_{0.80}Co_{0.15}Al_{0.05}O_2$ (NCA) electrode sheets with loading of 3 mAh cm^{-2} and $LiNi_{0.8}Mn_{0.1}Co_{0.1}O_2$ (NMC811) electrode sheets with loading of 4.5 mAh cm^{-2} are both kindly provided by Saft America, Inc. The ultrathin Li foil with 20 µm thickness is ordered from China Energy Lithium Company.

Characterizations. The Hitachi SU-70 field emission scanning electron microscope (SEM) was used for the morphology characterization. The Bruke Advance spectrometers (400 MHz) were performed for the NMR experiments. Inductively coupled plasma optical emission spectrometry (ICP-OES, Shimadzu ICPE-9000) was applied to determine the Mg content change in the electrolyte. The ToF-SIMS attached with a Ga^+ focused ion beam (FIB)/SEM (Tescan GAIA3) was employed to do the ion sputtering and elemental distributions analysis in depth (30 kV), of which the results were further analyzed by the ToF-SIMS Explorer and 3D images were generated using Dragonfly software, Version 2020.2 for Windows (Object Research Systems (ORS) Inc, Montreal, Canada). XPS was conducted on a Kratos AXIS 165 X-ray photoelectron spectrometer with the Mg Kα radiation, of which all the spectra were calibrated to the adventitious hydrocarbon peak at 284.6 eV and analyzed by the Casa XPS. All the electrodes after cycling were washed with EMC solvent and dried to remove the residual salts before characterizations.

Electrochemical measurements. The electrochemical performances of the Li metal batteries were all examined using 2032 type coin cell with 2 layers of the Celgard 2325 separator, which were assembled and disassembled in an Ar-filled glove box with O_2 and moisture content lower than 1 ppm. All the charge-discharge processes of Li||Cu and Li||NCM811 or NCA full cells were tested using the Arbin (BT2000, Arbin Instruments) or Landt (Wuhan Land) battery test station. For galvanostatic tests of full cells, NCM 811 cathodes with diameter of 12.7mm (1/2 inch) were coupled with 13mm Li anode with 20 µm thickness, of which the electrolyte amount was controlled of 25 µL. The long-term cycling stability was performed at C/5 charge and C/2 discharge after two formation cycles at C/10. Linear sweep voltammetry (LSV), cyclic voltammetry (CV), and electrochemical impedance spectroscopy (EIS) were carried out on the Gamry 1000E electrochemical workstation. All the electrochemical measurements were tested at room temperature.

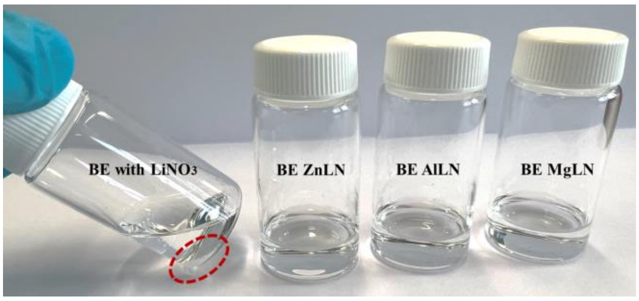

Figure S1. The digital images of 1.0M $LiPF_6$ in FEC-EMC electrolytes with only 25mmol $LiNO_3$ or with 25mmol X-125mmol $LiNO_3$ (X= $Zn(TFSI)_2$, $Al(OTf)_3$, $Mg(TFSI)_2$) additive.

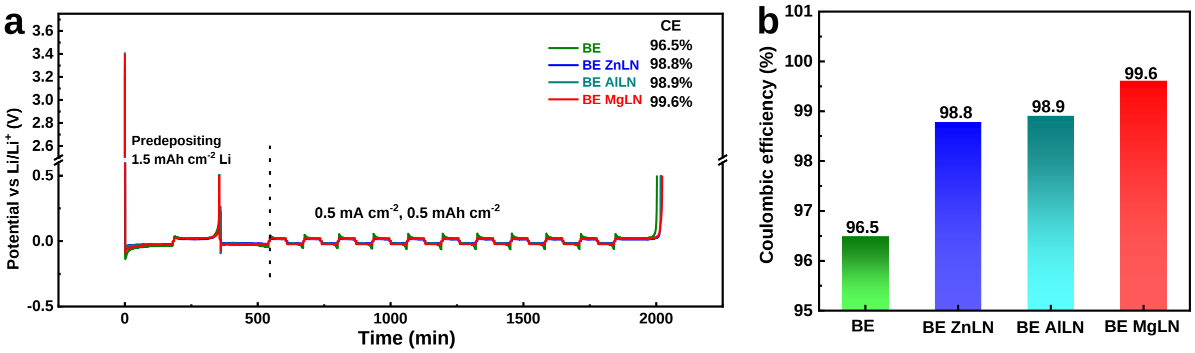

Figure S2. (a,b) The typical Li plating/stripping profile and the CE comparison in Li||Cu cells in BE with/without X/$LiNO_3$ (X= $Mg(TFSI)_2$, $Zn(TFSI)_2$, $Al(OTf)_3$) additive at a current density of 0.5 mA cm^{-2} and a capacity of 0.5 mAh cm^{-2} under 33% Li utilization.

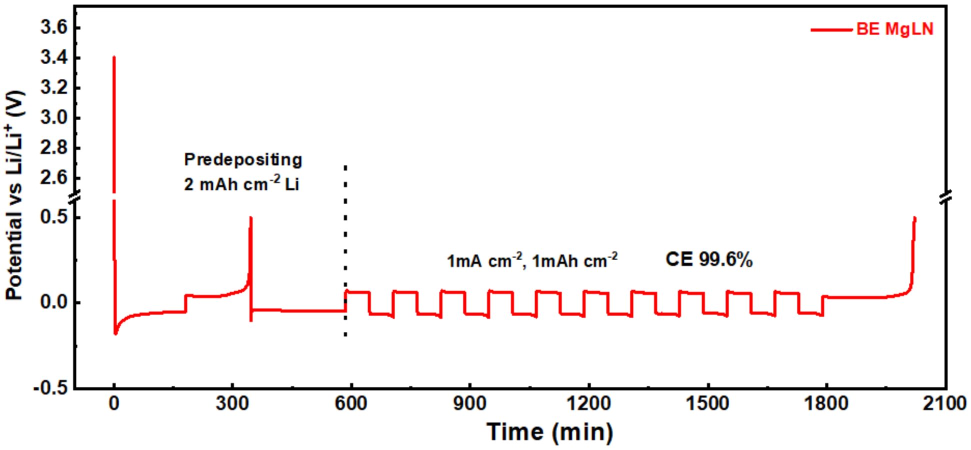

Figure S3. The typical Li plating/stripping profile and the CE value of Li||Cu cell in BE MgLN at 1mA cm^{-2} with a capacity of 1 mAh cm^{-2}.

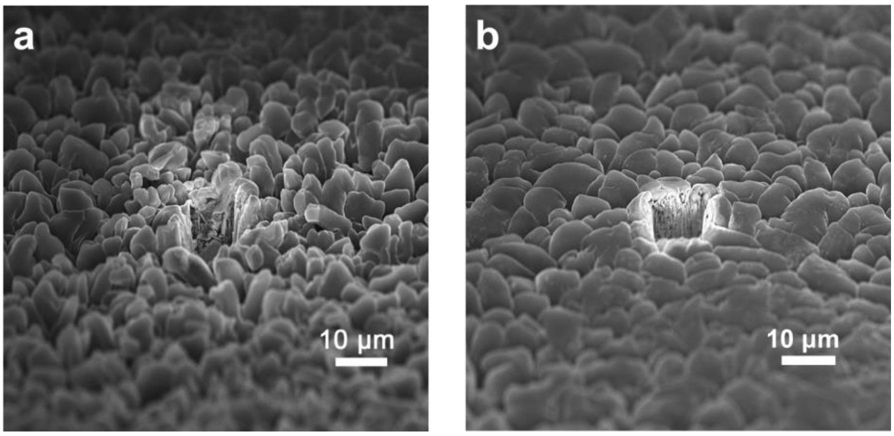

Figure S4. The SEM images of the plated Li metal in (a) BE and (b) BE MgLN, of which the crater was sputtered by the Ga^+ ion beam with a 10μm x 10μm area.

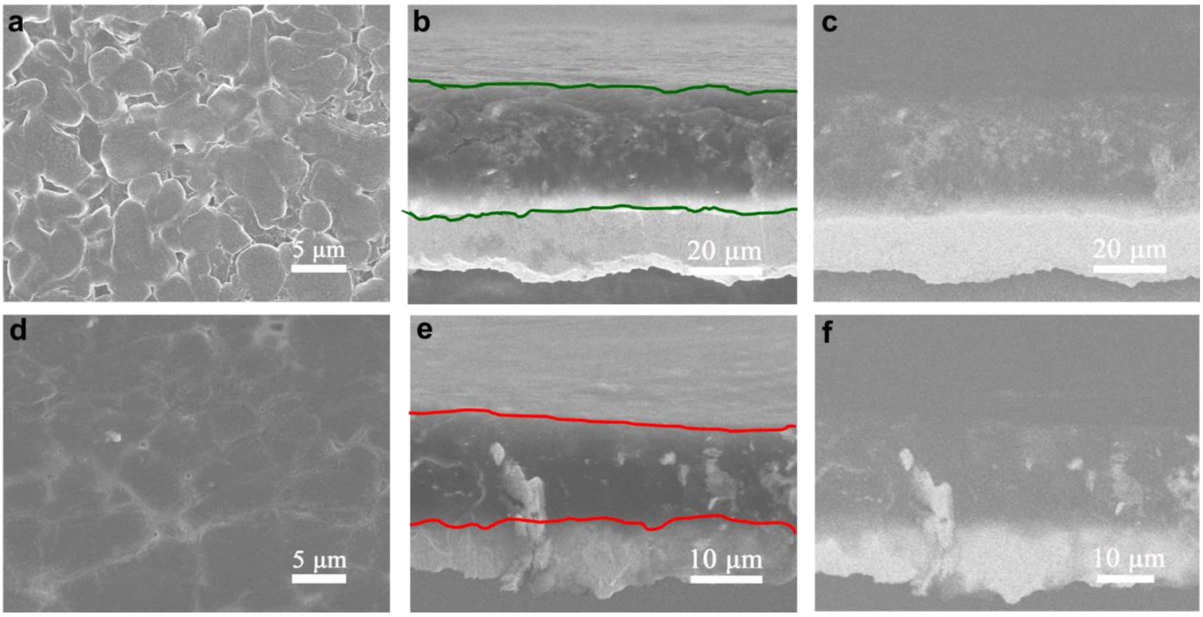

Figure S5. The typical top-view and cross-section SEM images of the 2mAh cm^{-2} Li plated in (a,b) BE and (d,e) BE MgLN, (c, f) the corresponding backscattered electron images.

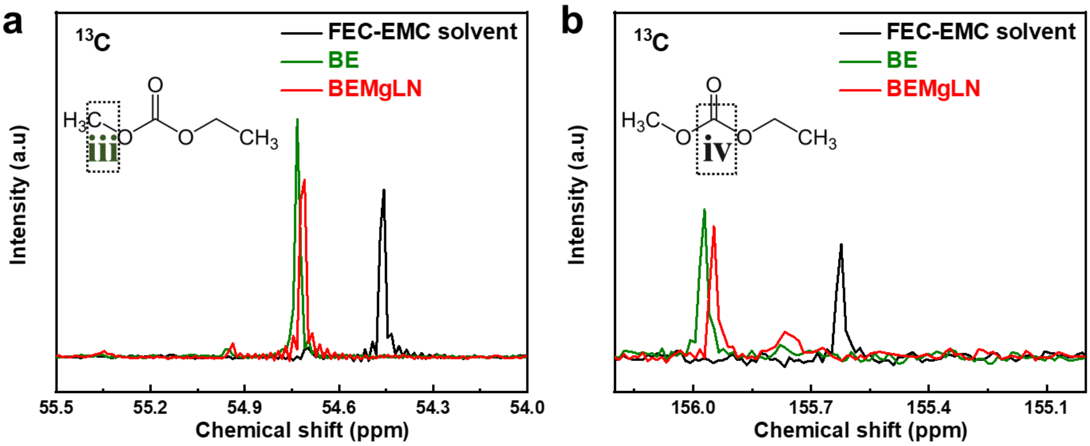

Figure S6. The ^{13}C-NMR spectra of EMC in the various electrolytes and FEC-EMC (3:7 by vol.) solution in other ranges.

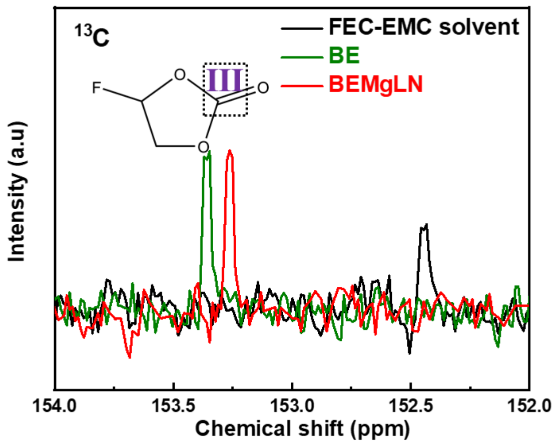

Figure S7. The ^{13}C-NMR spectra of FEC in the various electrolytes and FEC-EMC (3:7 by vol.) solution in other ranges.

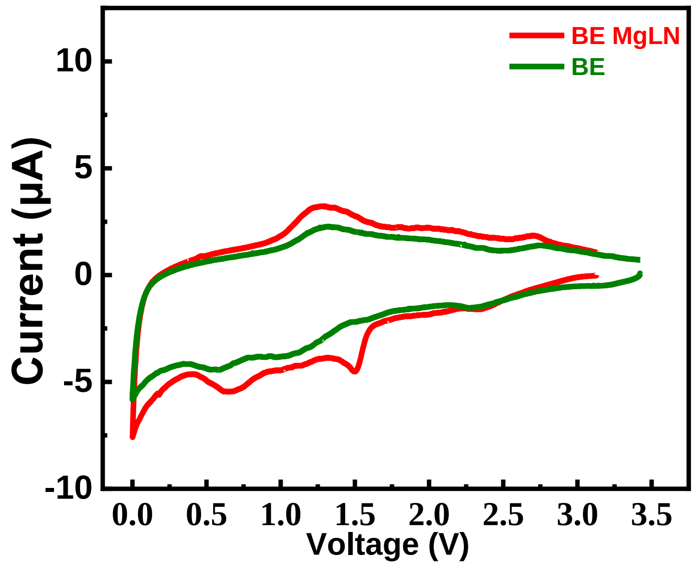

Figure S8. Typical CV curves of Li||Cu cells with different electrolytes, which was scanned at a rate of 0.1 mV s^{-1}.

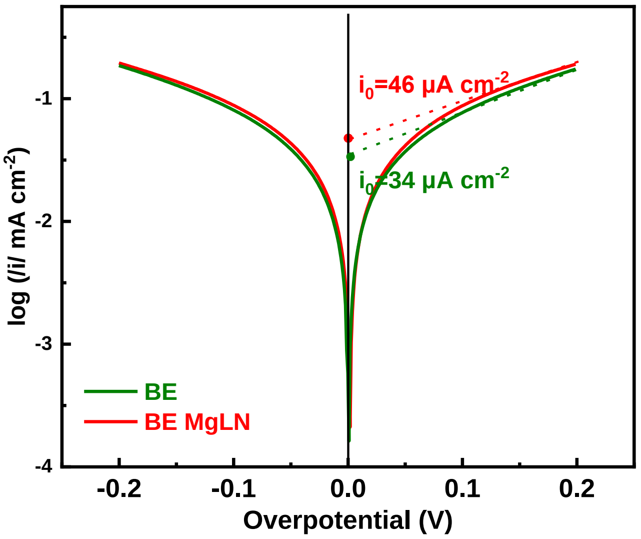

Figure S9. The Tafel plots obtained from a cyclic voltammetry test in Li-Li cells with different electrolytes, which is scanned at a rate of 1.0 mVs^{-1} between -0.2 to 0.2 V.

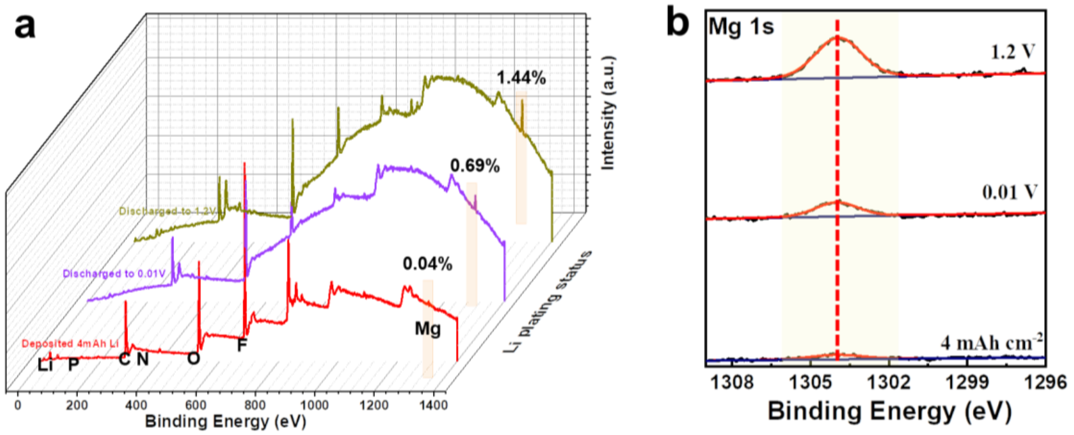

Figure S10. (a) Wide-spectrum XPS survey and (b) the specific Mg 1s spectra of the Cu electrode interphase in Li||Cu cells at different Li plating status.

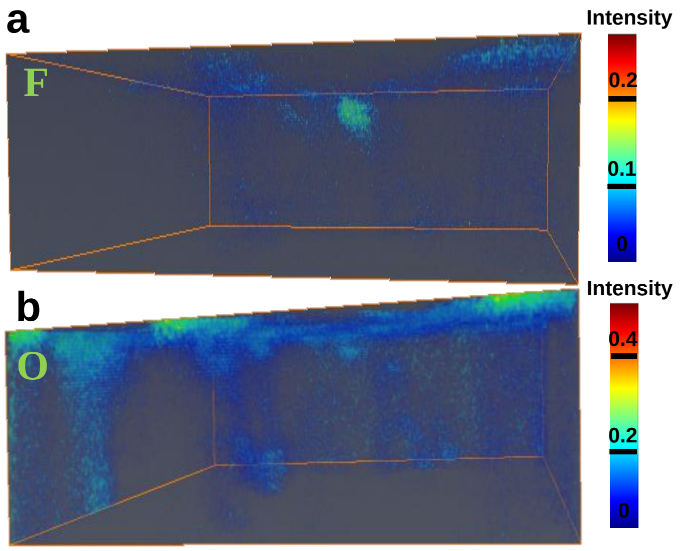

Figure S11. 3D reconstruction of the elemental F and O distribution of the electrochemically deposited Li surface in BE.

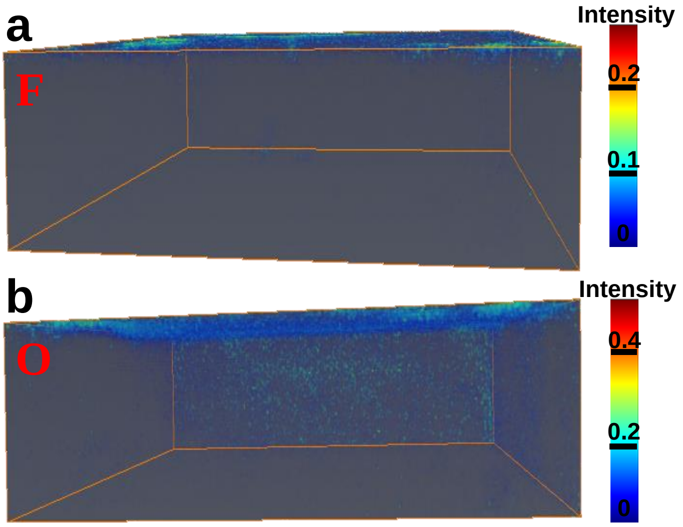

Figure S12. 3D reconstruction of the elemental F and O distribution of the electrochemically deposited Li surface in BE MgLN.

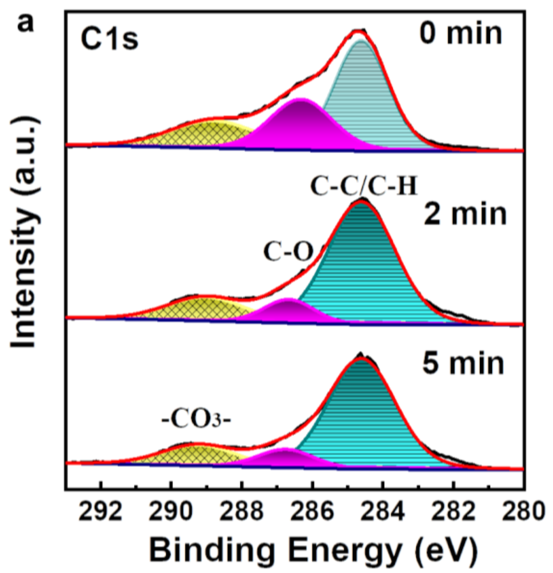

Figure S13. The in-depth C1s spectra of the SEI layer formed in (a) BE and (b) BE MgLN electrolytes by the XPS measurement.

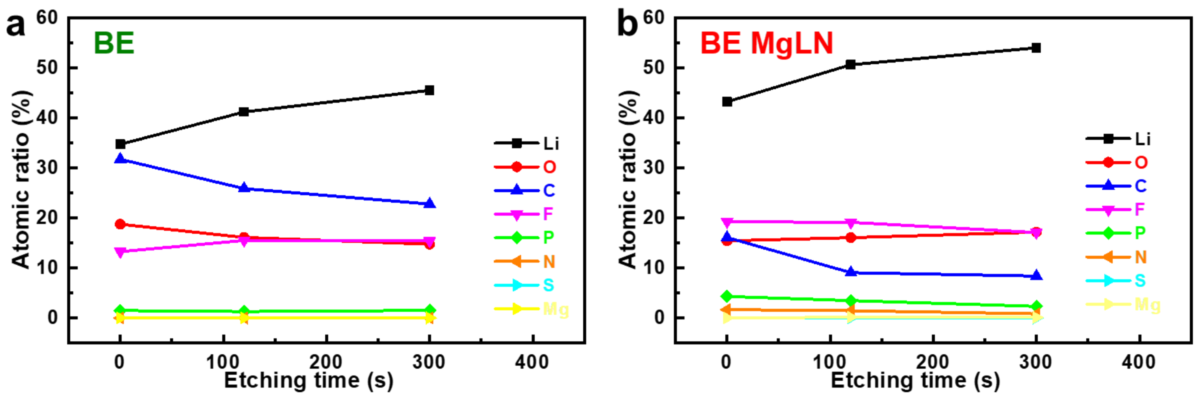

Figure S14. The in-depth atomic composition ratios of the SEI layer formed in (a) BE and (b) BE MgLN.

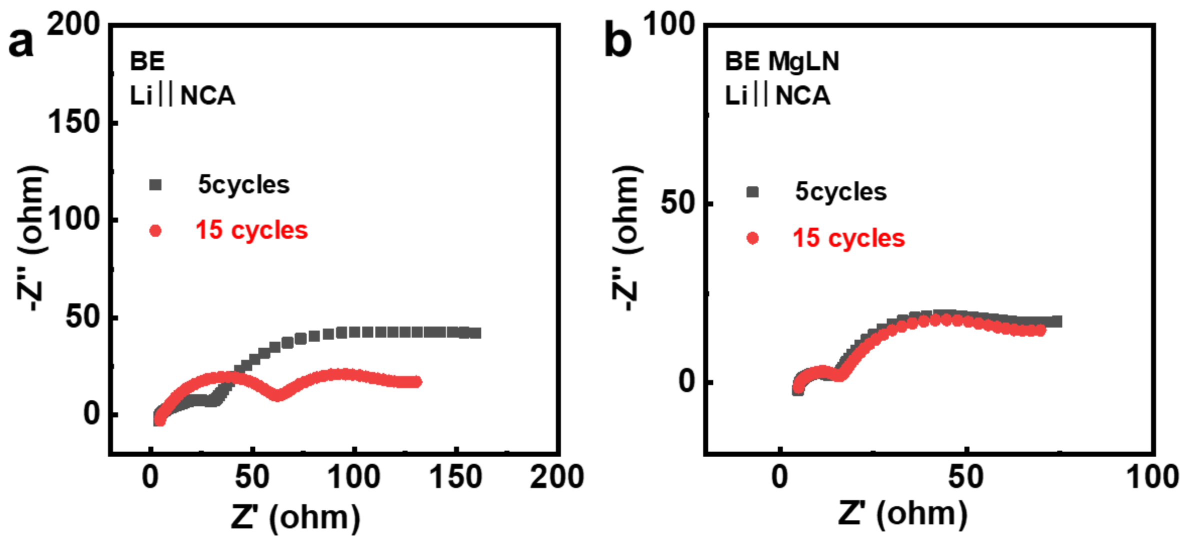

Figure S15. Nyquist plots the Li||NCA cells after 5 and 15 cycles in (a) BE and (b) BE MgLN electrolytes.

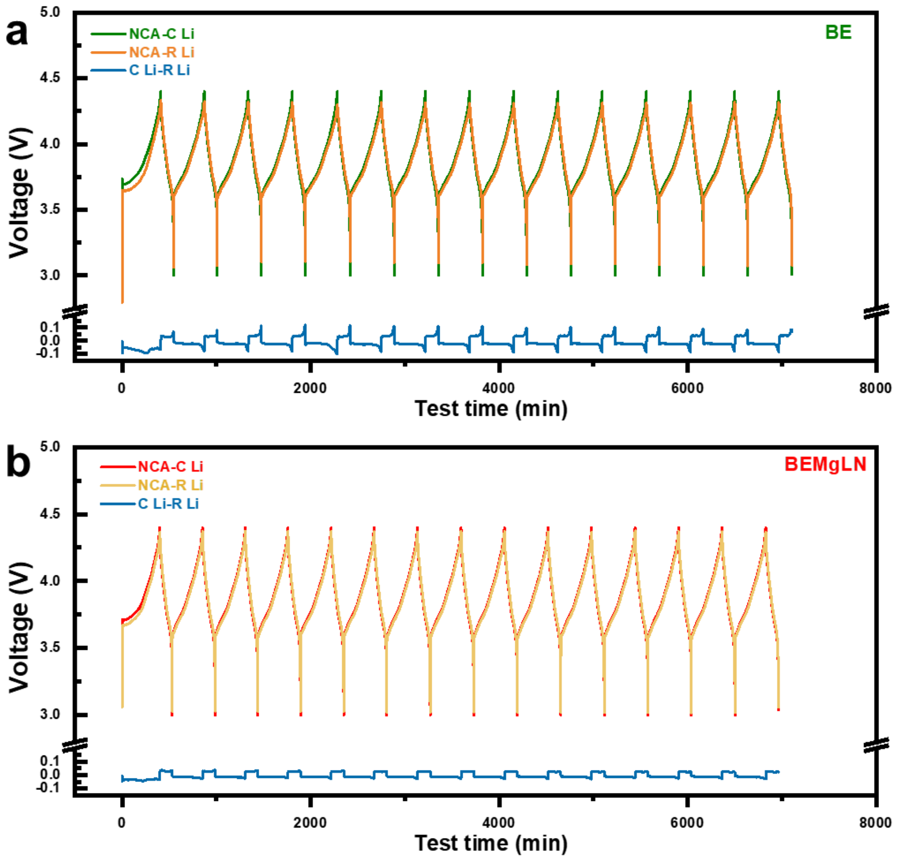

Figure S16. The full-range voltage-time profile of the three-electrode cell with working NCA cathode, counter Li (C Li) anode and reference Li (R Li) electrode along the cycling. 0.2C charge/0.5C discharge.

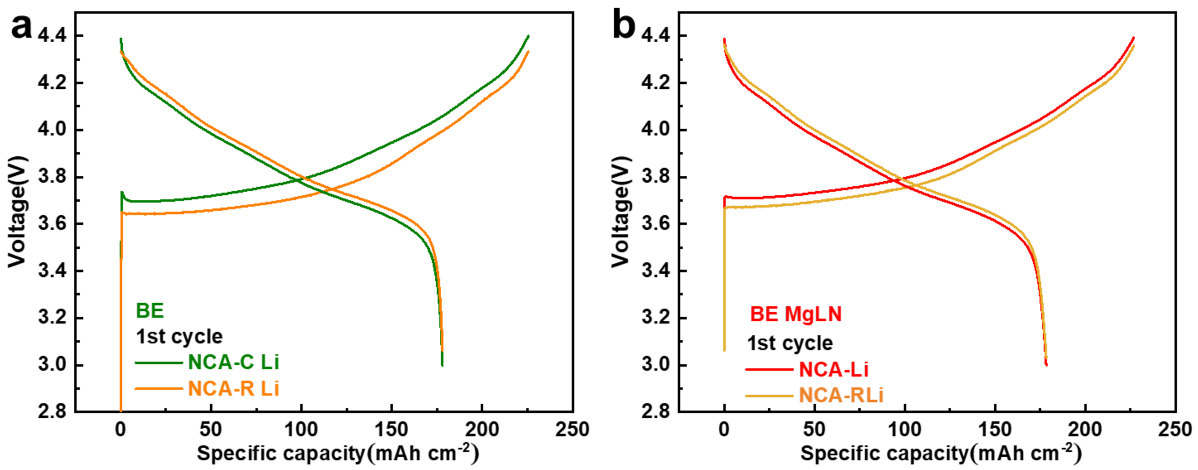

Figure S17. The first charging/discharging profile of NCA cathode coupled with counter Li anode or reference Li electrode in the three-electrode Li||NCA batteries.

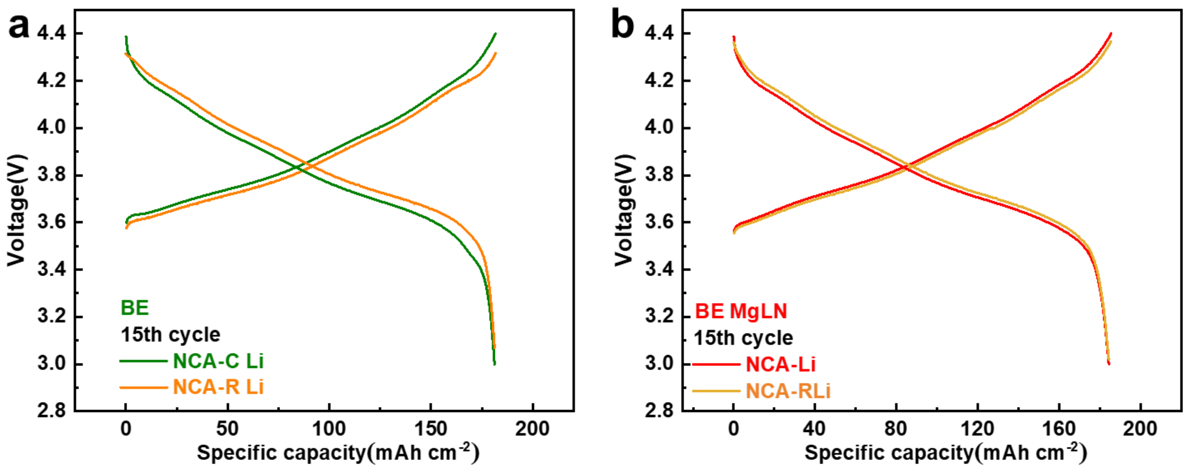

Figure S18. The 15th charging/discharging profile of NCA cathode coupled with counter Li anode or reference Li electrode in the three-electrode Li||NCA batteries.

Electrolytes	Current density (mA cm^{-2})	Capacity (mAh cm^{-2})	Testing cycles	CE	Reference
1M $LiPF_6$-125 mmol $LiNO_3$-25 mmol $Mg(TFSI)_2$ in FEC-EMC, named as BE MgLN	0.5	2.5	10	99.5%	This work
		4.5	10	99.7%	
	1	1	10	99.6%	
1M $LiPF_6$ FEC/DMC (1:4 by vol.)	0.5	1	38	98.5%	[1]
1M $LiPF_6$ in EC/DEC (1:1 by vol.) with 0.15M LiDFP	0.4	0.5	100	95.2	[2]
1M $LiPF_6$ EC/DEC(1:1 by vol.) with 0.2 wt%CuF_2 and 1.0wt% $LiNO_3$	0.5	0.5	10	98.1%	[3]
1M $LiPF_6$ EC/DEC (1:1 by vol.) with 5vol% FEC additive	0.1	0.5	100	98%	[4]
	0.5	0.5	100	95%	
0.5M $LiPF_6$ EC/DEC (1:1 by vol.) with $LiNO_3$@PVDF-HFP buffer layer	1	1	200	98.1%	[5]
0.8M $LiPF_6$ FEC/DMC (1:4 by vol.) +5wt% 4M $LiNO_3$-DMSO)	1	1	10	99.55%	[6]
0.6 m LiTFSI and 0.4 m LiBOB in EC-EMC (7:3 by wt.) +0.6wt% $LiPF_6$+2.0wt%VC and 2.0 wt% FEC	0.5	0.5	11	98.1%	[7]
1M $LiPF_6$ in EC/DMC (1:2 by vol.) with 2%TTFEB	0.1	0.5	100	99%	[8]
2.2m LiFSI in TEP +5vol%FEC+0.05m LiBOB	0.2	1	Between 0-140	99%	[9]
10M LiFSI EC-DMC (1:1 by vol.)	0.2	1	Between 100-240	99.3%	[10]
7M LiFSI FEC	0.25	0.5	Between 0-100	97.7%	[11]
			Between 300-400	99.6%	
4M LiFSI-DME	0.2	1	500	99.1%	[12]
	1	1	500	98.5%	
1M $LiPF_6$ FEC/FEMC/HFE（2:6:2 by wt.）	0.2	1	Within 50	99.2%	[13]
1.2M LiFSI TEP-BTFE (1:3 by mol)	0.5	1	9	99.2%	[14]
	3	1	9	98.5	
1.2M LiFSI in DMC-BTFE (1:2 by mol)	0.5	1	10	99.3%	[15]
LiFSI-DMC-TTE (1:1.5:1.5 by mol)	1	1	400	98.6%	[16]
LiFSI-DME-TTE (1:1.2:3 by mol)	0.5	1	220	99.1%	[17]
1M LiFSI in TFEO-DME (1:9 by wt.)	0.5	1	10	99.5%	[18]

Supplementary Table 1. Coulombic efficiency comparison of the Li metal anode in Li||Cu cells with our designed electrolyte with available data in other electrolytes.

Electrolytes	Li metal anode	Cathode	N/P ratio	Cycling conditions	Performance	Reference
1M $LiPF_6$-125 mmol $LiNO_3$-25 mmol $Mg(TFSI)_2$ in FEC-EMC, named as BE MgLN	20 μm Li	3.0 mAh cm^{-2} NCA	2.37	C/5 charge, C/2 discharge, 2.7-4.4V	87.4% after 200 cycles	This work
		4.5 mAh cm^{-2} NMC811	1.92	C/5 charge, C/2 discharge, 2.7-4.4V	84.6% after 200 cycles	
1M $LiPF_6$ FEC/DMC (1:4 by vol.)	50 μm Li	3.3 mAh cm^{-2} NMC622	4.12	0.15C, 2.8-4.3 V	Around 90% after 90 cycles	[1]
1M $LiPF_6$ EC/DEC(1:1 by vol.) with 0.2 wt% CuF_2 and 1.0wt% $LiNO_3$	Thick Li	2.5 mAh cm^{-2} NCA	/	0.5 C, 3.0-4.3 V	53% after 300 cycles	[3]
1M $LiPF_6$ EC/DEC (1:1 by vol.) with 5vol% FEC additive	Thick Li	1.9 mAh cm^{-2} NMC532	/	1.0 C, 3.0-4.3 V	65% after 100 cycles	[4]
0.5M $LiPF_6$ EC/DEC (1:1 by vol.) with $LiNO_3$@PVDF-HFP buffer layer	42 μm Li	1.2 mAh cm^{-2} NMC111	8.22	0.8C, 2.7-4.3 V	Around 80% after 100 cycles	[5]
0.8M $LiPF_6$ FEC/DMC (1:4 by vol.) +5wt% 4M $LiNO_3$-DMSO)	50 μm Li	2.5 mAh cm^{-2} NMC811	5.12	0.5C, 2.7-4.4V	75% after 200 cycles	[6]
0.6 m LiTFSI and 0.4 m LiBOB in EC-EMC (7:3 by wt.) +0.6wt% $LiPF_6$+2.0wt%VC and 2.0 wt% FEC	Thick Li	1.75 mAh cm^{-2} NMC442	/	1C, 2.7-4.3V	97.1% after 500 cycles	[7]
10M LiFSI EC-DMC (1:1 by vol.)	Thick Li	13 mg cm^{-2} NMC442	/	2.7-4.6V	86% after 100 cycles, which	[10]
7M LiFSI FEC	9.5 μm Li (electro-plated)	1.83 mAh cm^{-2} LNMO	2.09	0.36C, 3.4-5V	Around 70% after 140 cycles	[11]
1M $LiPF_6$ FEC/FEMC/HFE（2:6:2 by wt.）	9.5 μm Li (electro-plated)	2 mAh cm^{-2} NMC811	2	0.5C, 2.7-4.4V	Nearly 100% after 120 cycles	[13]
1.2M LiFSI in DMC-BTFE (1:2 by mol)	Thick Li	2 mAh cm^{-2} NMC622	/	1C, 2.7-4.3V	95% after 300 cycles	[15]
LiFSI-DMC-TTE (1:1.5:1.5 by mol)	Thick Li	1.5 mAh cm^{-2} NMC811	/	0.5C, 2.7-4.6V	93.5% after 100 cycles	[16]
LiFSI-DME-TTE (1:1.2:3 by mol)	50 μm Li	4.2 mAh cm^{-2} NMC811	3.45	0.33C, 2.8-4.4V	80% after 155 cycles	[17]
1M LiFSI in TFEO-DME (1:9 by wt.)	50 μm Li	1.5 mAh cm^{-2} NMC811	7.87	0.33C, 2.8-4.4V	80% after 300 cycles	[18]

Supplementary Table 2. Full cell performance comparison with our designed electrolyte with available data in other electrolytes.

References

[1] E. Markevich, G. Salitra, F. Chesneau, M. Schmidt, D. Aurbach, *ACS Energy Lett.* **2017**, *2*, 1321-1326.

[2] P. Shi, L. Zhang, H. Xiang, X. Liang, Y. Sun, W. Xu, *ACS Appl. Mater. Interfaces* **2018**, *10*, 22201-22209.

[3] C. Yan, Y. X. Yao, X. Chen, X. B. Cheng, X. Q. Zhang, J. Q. Huang, Q. Zhang, *Angew. Chem. Int. Ed.* **2018**, *57*, 14055-14059.

[4] X. Q. Zhang, X. B. Cheng, X. Chen, C. Yan, Q. Zhang, *Adv. Funct. Mater.* **2017**, *27*, 1605989.

[5] Y. Liu, D. Lin, Y. Li, G. Chen, A. Pei, O. Nix, Y. Li, Y. Cui, *Nat. Commun.* **2018**, *9*, 3656.

[6] S. Liu, X. Ji, N. Piao, J. Chen, N. Eidson, J. Xu, P. Wang, L. Chen, J. Zhang, T. Deng, S. Hou, T. Jin, H. Wan, J. Li, J. Tu, C. Wang, *Angew. Chem. Int. Ed.* **2020**, *60*, 3661-3671.

[7] J. Zheng, M. H. Engelhard, D. Mei, S. Jiao, B. J. Polzin, J. G. Zhang, W. Xu, *Nat. Energy* **2017**, *2*, 17012.

[8] Y. Ma, Z. Zhou, C. Li, L. Wang, Y. Wang, X. Cheng, P. Zuo, C. Du, H. Huo, Y. Gao, G. Yin, *Energy Storage Mater.* **2018**, *11*, 197-204.

[9] Z. Zeng, V. Murugesan, K. S. Han, X. Jiang, Y. Cao, L. Xiao, X. Ai, H. Yang, J.-G. Zhang, M. L. Sushko, J. Liu, *Nat. Energy* **2018**, *3*, 674-681.

[10] X. Fan, L. Chen, X. Ji, T. Deng, S. Hou, J. Chen, J. Zheng, F. Wang, J. Jiang, K. Xu, C. Wang, *Chem* **2018**, *4*, 174-185.

[11] L. Suo, W. Xue, M. Gobet, S. G. Greenbaum, C. Wang, Y. Chen, W. Yang, Y. Li, J. Li, *Proc. Natl. Acad. Sci. U. S. A.* **2018**, *115*, 1156-1161.

[12] J. Qian, W. A. Henderson, W. Xu, P. Bhattacharya, M. Engelhard, O. Borodin, J. G. Zhang, *Nat. Commun.* **2015**, *6*, 6362.

[13] X. Fan, L. Chen, O. Borodin, X. Ji, J. Chen, S. Hou, T. Deng, J. Zheng, C. Yang, S. C. Liou, K. Amine, K. Xu, C. Wang, *Nat. Nanotechnol.* **2018**, *13*, 715-722.

[14] C. Niu, H. Lee, S. Chen, Q. Li, J. Du, W. Xu, J.-G. Zhang, M. S. Whittingham, J. Xiao, J. Liu, *Nat. Energy* **2019**, *4*, 551-559.

[15] S. Chen, J. Zheng, D. Mei, K. S. Han, M. H. Engelhard, W. Zhao, W. Xu, J. Liu, J. G. Zhang, *Adv. Mater.* **2018**, *30*, 1706102.

[16] N. Piao, X. Ji, H. Xu, X. Fan, L. Chen, S. Liu, M. N. Garaga, S. G. Greenbaum, L. Wang, C. Wang, X. He, *Adv. Energy Mater.* **2020**, *10*, 1903568.

[17] X. Ren, L. Zou, X. Cao, M. H. Engelhard, W. Liu, S. D. Burton, H. Lee, C. Niu, B. E. Matthews, Z. Zhu, C. Wang, B. W. Arey, J. Xiao, J. Liu, J.-G. Zhang, W. Xu, *Joule* **2019**, *3*, 1662-1676.

[18] X. Cao, X. Ren, L. Zou, M. H. Engelhard, W. Huang, H. Wang, B. E. Matthews, H. Lee, C. Niu, B. W. Arey, Y. Cui, C. Wang, J. Xiao, J. Liu, W. Xu, J.-G. Zhang, *Nat. Energy* **2019**, *4*, 796-805.
